# Cubozoan Sting-Site Seawater Rinse, Scraping, and Ice Can Increase Venom Load: Upending Current First Aid Recommendations

**DOI:** 10.3390/toxins9030105

**Published:** 2017-03-15

**Authors:** Angel Anne Yanagihara, Christie L. Wilcox

**Affiliations:** 1 Department of Tropical Medicine, Medical Microbiology and Pharmacology, John A. Burns School of Medicine, University of Hawaii at Mānoa, Honolulu, HI 96822, USA; wilcoxcl@hawaii.edu; 2 Békésy Laboratory of Neurobiology, Pacific Biosciences Research Center, University of Hawaii at Mānoa, Honolulu, HI 96822, USA

**Keywords:** first aid, marine envenomation, box jelly, *Alatina alata*, *Chironex fleckeri*, jellyfish, hot water immersion, heat treatment, shaving

## Abstract

Cnidarian envenomations are the leading cause of severe and lethal human sting injuries from marine life. The total amount of venom discharged into sting-site tissues, sometimes referred to as “venom load”, has been previously shown to correlate with tentacle contact length and sequelae severity. Since <1% of cnidae discharge upon initial tentacle contact, effective and safe removal of adherent tentacles is of paramount importance in the management of life-threatening cubozoan stings. We evaluated whether common rinse solutions or scraping increased venom load as measured in a direct functional assay of venom activity (hemolysis). Scraping significantly increased hemolysis by increasing cnidae discharge. For *Alatina alata*, increases did not occur if the tentacles were first doused with vinegar or if heat was applied. However, in *Chironex fleckeri*, vinegar dousing and heat treatment were less effective, and the best outcomes occurred with the use of venom-inhibiting technologies (Sting No More^®^ products). Seawater rinsing, considered a “no-harm” alternative, significantly increased venom load. The application of ice severely exacerbated *A. alata* stings, but had a less pronounced effect on *C. fleckeri* stings, while heat application markedly reduced hemolysis for both species. Our results do not support scraping or seawater rinsing to remove adherent tentacles.

## 1. Introduction

Box jellyfish stings are an increasing public health problem, causing more deaths annually than shark attacks worldwide [1,2,3,4]. Envenomations can range in severity from mild sting-site pain to loss of life within minutes [4,5,6,7]. Sting severity varies based on several factors, the most important of which are the species involved and the dose of venom delivered [8,9]. Studies have shown that the outcomes also vary based on first-aid and clinical-care measures [10,11,12,13]. The activity of venom, which is already deposited into the tissue of the victim at the sting site, can be markedly reduced by using venom-inhibiting treatments, such as hot-water immersion [10,11,14,15] and dermal-permeating inhibitors [15]. Since only <1% of tentacle cnidae discharge upon initial contact with human skin [16], ineffective procedures for removing still-adherent live tentacles have the potential to greatly worsen sting outcomes by increasing the initial venom load.

While the preponderance of evidence supports the safe, effective, and advantageous use of vinegar as a rinse solution [16,17,18,19] and hot-water immersion or hot packs for pain relief after vinegar rinsing [14], suggestions for appropriate first aid in the absence of vinegar vary greatly. One of the most commonly repeated recommendations is the use of scraping with a credit card or razor to remove tentacles and/or undischarged cnidae [20,21,22,23,24,25,26,27,28,29,30,31,32,33,34,35,36,37,38,39,40]. This is perhaps a surprising suggestion given that it is known that pressure can cause both undischarged cnidae, packed on the surface of the tentacles, as well as intact cnidae shed from the tentacle and left on the skin, to fire [41]; indeed, some medical professionals explicitly recommend against sting-site scraping [42] (p. 279). To our knowledge, the safety and efficacy of scraping has not previously been experimentally examined. Thus, we sought to compare the effects of scraping alone and in combination with other first aids in two cubozoan species, *Alatina alata* (Hawaiian box jellyfish, family: Carybdeidae) and *Chironex fleckeri* (Australian sea wasp, family: Chirodropidae).

In part, the lack of evaluation of scraping is due to the methods of experimental examination used to inform clinical management, which do not directly allow for the examination of mechanical procedures like scraping. While there have been a few clinical studies, there has been a pervasive assumption that solution-based assays, such as the tentacle solution assay (TSA), are reliable predictors of clinical outcomes. The TSA, which measures cnidae discharge in the presence of tested solutions with or without a secondary cnidae stimulus (chemical or physical), is often used to evaluate removal aids, although direct correlations between the assay results and clinical outcomes have not been well established. There is also a lack of rigorous correlation between acute findings like pain and redness and long-term clinical outcomes, further hampering clarity in evaluating first-aid measures.

TSA studies have shown vinegar to be a potent, irreversible inhibitor of cubozoan cnidae discharge [16,17]. Its use is supported by case studies [43], but its effects on acute indicators, such as pain and redness, in human cubozoan sting trials are mixed [44,45,46]. There is moderate evidence that solutions that induce significant discharge in the TSA, such as ethanol, worsen pain [44,45], but their effects on overall outcomes are unclear. Seawater does not induce cnidae discharge in the TSA, but it does not inhibit the subsequent potential discharge of cnidae, either [16,17,18,44,45]. It is interesting to note that although seawater is often used as a control for other rinse solutions in clinical studies, no studies to our knowledge have specifically compared the effects of rinsing with seawater against any kind of no-rinse control (such as where tentacles are removed by plucking instead), so whether it or other “inert” solutions are appropriate to use as rinses has never been rigorously studied; and yet, seawater rinse is the second-most recommended method of removal, especially when vinegar is not available [20,21,25,26,29,30,31,32,33,34,35,36,37,38]. Thus, we sought to compare the effects of rinsing with different solutions (seawater, freshwater, ethanol, Sting No More^®^ Spray, and vinegar) to removal by scraping in a functional assay of venom activity utilizing blood agarose [16]. By comparing the rinse solutions and scraping, as well as to a no-rinse control (plucking tentacles with tweezers), we aimed to evaluate the relative efficacy of these removal methods. We further sought to quantify the effect of scraping on venom delivery when preceded by vinegar dousing or followed by the application of heat.

In addition, we tested two scraping aids—a baking soda “slurry” and shaving cream—as well as dilutions of vinegar on cnidae discharge inhibition using the TSA. The effects of a baking soda “slurry” in the TSA has only been examined in two species of cubozoan: *Carybdea rastoni*, where it was reported to inhibit chemically induced discharge [18], and *Morbakka*, where it did not induce discharge but failed to inhibit it [47]. However, the details of the makeup of the baking soda solutions were not reported, and since vinegar also inhibited discharge, the authors recommended it preferentially in both cases. We could find no clinical studies evaluating baking soda for cubozoan stings. Shaving cream has never been evaluated for its effects on cnidae or its clinical effects, and yet it is commonly recommended in conjunction with scraping [38]. Specifically, our aim was to examine whether baking soda slurries or shaving cream inhibited pressure-induced cnidae discharge, as scraping or rubbing would elicit mechanical discharge rather than chemical discharge of cnidae, as well as whether diluted vinegar was capable of the same irreversible inhibition of discharge as undiluted vinegar.

## 2. Results

### 2.1. Effect of Scraping on Cnidae Removal and Discharge on Isolated Skin

**Figure 1:**
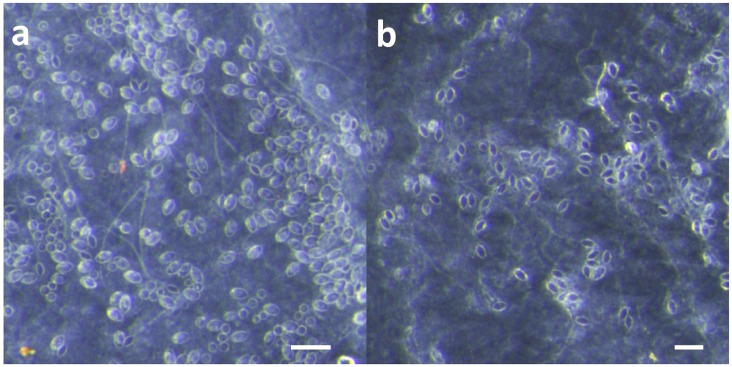
Comparison of cnidae remaining on model skin after removing the tentacle by (**a**) pulling with tweezers and (**b**) scraping with a credit card (*n* = 3 replicate tentacle sections for each removal method; cnidae counts from 3 images per tentacle were averaged). Scraped areas had fewer total cnidae (263 ± 19 per mm^2^ versus 505 ± 39 per mm^2^), but more of them were discharged (85.1% ± 2.6% versus 27.9% ± 1.3%), leading to nearly double the number of discharged cnidae per mm^2^ of skin (211 ± 9 per mm^2^ versus 96 ± 40 per mm^2^). Scale bars shown represent 100 μm.

To determine if scraping effectively removes cnidae or causes further cnidae discharge, we applied live *A. alata* tentacles to a skin model made from porcine intestine (a part of the “TSBAA” (tentacle skin blood agarose assay) model outlined in [16]) and either pulled or scraped the tentacles off after a 10 min sting. The skin was then examined microscopically to determine the number of adherent cnidae per unit area and whether they were discharged or undischarged (Figure 1). About half as many cnidae—263 ± 19 versus 505 ± 39—remained on the skin after scraping as opposed to pulling the tentacles off with tweezers (two-tailed *t*-test t(4) = 5.559, *p* = 0.005). However, the percentage of cnidae that were discharged was significantly higher when the tentacles were scraped (85.1% ± 2.6% compared to 27.9% ± 1.3%; two-tailed *t*-test t(4) = 19.67, *p* < 0.001), which resulted in more discharged cnidae per unit area (211 ± 9 per mm^2^ for scraped versus only 96 ± 40 per mm^2^ for pulled, two-tailed *t*-test t(4) = 2.81, *p* = 0.048).

### 2.2. Effect of Adherent Tentacle Removal by Scraping

Removal of adherent tentacles is one of the most important steps in first-aid management of cnidarian stings. With that in mind, we sought to compare removal by scraping or pulling off with tweezers to other commonly used or previously recommended solutions for rinsing off tentacles. We set up a previously described TSBAA (see Methods, below) [16], with the modification that instead of simply removing tentacles by pulling them off, stinging tentacles were either sprayed off or scraped off. Tentacles pulled off with tweezers were used as a baseline.

The removal method had a significant impact on the size of the hemolytic zone for stings from both *A. alata* and *C. fleckeri* at both single time points (one-way ANOVAs, *p* < 0.0001) and over a time course (two-way ANOVAs, *p* < 0.001 for removal method). For *A. alata*, scraping was significantly worse than all other removal methods at all time points (post hoc Fisher’s least significant difference (LSD), *p* < 0.05 for all comparisons). Rinsing with ethanol was not effective at removing the tentacles; rather than being rinsed away, massive cnidae discharge occurred and the tentacles became even more adherent and had to be manually pulled off.

**Figure 2:**
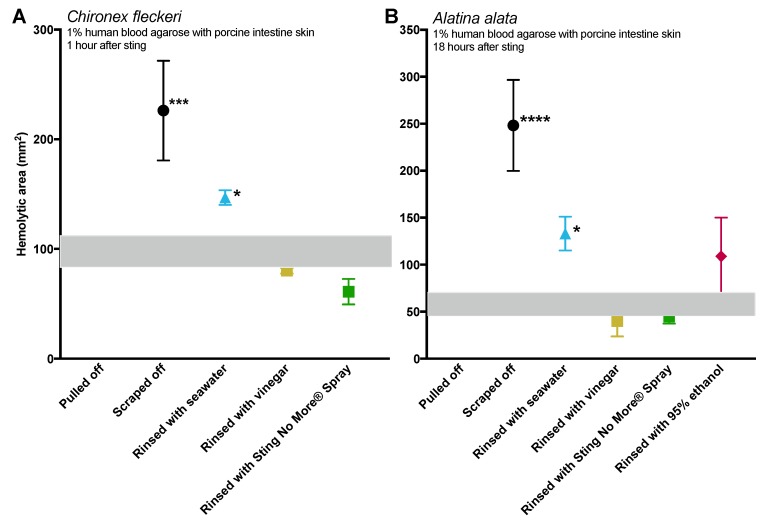

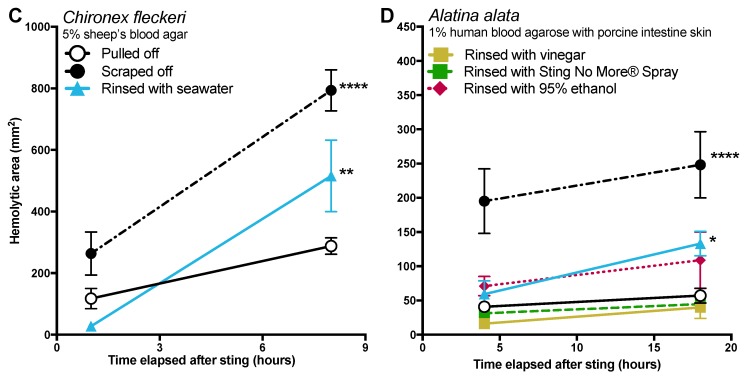
Size of venom-induced hemolytic zone using different removal methods; grey zone indicates mean ± standard error of pulled off tentacles (used as a baseline). Experiments examined effects at either single time points (**A**,**B**) or over a time course (**C**,**D**), and in either *C. fleckeri* (**A**,**B**) or *A. alata* (**B**,**D**). Sample sizes for each were 3 replicate tentacle sections per test measure for (**A**) and 6 for (**B**,**C**,**D**). For the single time point studies, significant differences between the removal methods were found for both species (one-way ANOVA; *p* < 0.0001 for both); significant post hoc Fisher’s least significant difference (LSD) multiple comparisons to pulled off tentacles are denoted with asterisks: * *p* < 0.05, ** *p* < 0.01, *** *p* < 0.001, **** *p* < 0.0001. The hemolytic zone size of scraped tentacles was significantly greater than all other removal strategies (post hoc Fisher’s LSD multiple comparisons, all *p* < 0.001); rinsing with seawater also significantly increased hemolytic zone size (*p* = 0.0403 and 0.0331 for *C. fleckeri* and *A. alata*, respectively). Over a time course, significant differences between the removal methods were also found for both species (two-way repeated measures ANOVA). The removal method accounted for 54.75% of the variation (*p* < 0.0001) and 47.09% (*p* < 0.0001) for *A. alata* and *C. fleckeri*, respectively; time after sting accounted for 3.76% (*p* = 0.0103) and 19.66% (*p* < 0.0001), and for *C. fleckeri*, the interaction between the two was significant and accounted for 16.75% (*p* < 0.0001). The hemolytic zone size of scraped tentacles was significantly greater than all other removal strategies (post hoc Fisher’s LSD multiple comparisons, all *p* < 0.05); rinsing with seawater also significantly increased hemolytic zone size (*p* = 0.016 and 0.001 for *A. alata* and *C. fleckeri*, respectively, at latest time point). Ethanol, which was unable to remove the tentacles on its own, led to moderate but nonsignificant increases in lysis for *A. alata* (*p* = 0.0973).

Removal methods tended to have similar patterns of effects for *C. fleckeri* (Figure 2), however, tentacle removal was markedly more difficult for the chirodropid. Seawater and vinegar failed to rinse away adherent tentacles, which instead had to be plucked off after rinse attempts failed. Only Sting No More^®^ Spray effectively lifted adherent tentacles so they could be rinsed away.

**Figure 3:**
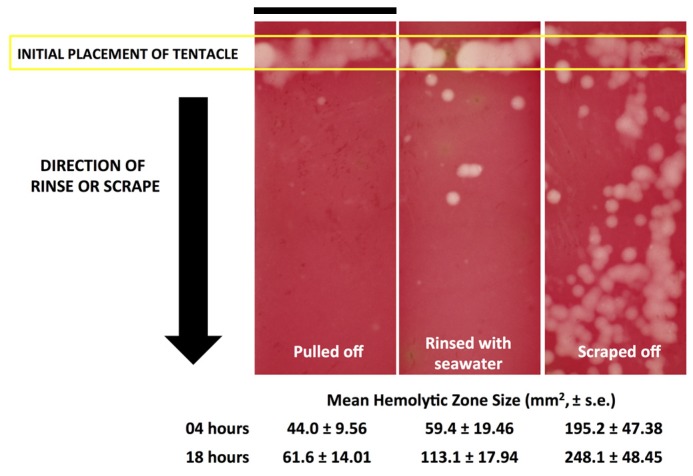
Comparison of hemolytic zones (white or light pink) from pulled, rinsed with seawater, and scraped tentacles in the blood agarose model 18 h post-sting. Scraping of tentacles without inhibiting cnidae discharge first led to clear continued stinging in the direction of the scrape, leading to an overall increase in venom injection. Scale bar shown over left most slide represents 20 mm.

In both species, contrary to the prevailing belief in the field, rinsing with seawater was not innocuous. Liberal seawater rinsing dispersed the intact cnidae over an area beyond the original tentacle contact site and resulted in subsequent discharge with hemolysis (Figure 2). Examination of agarose sting patterns revealed that seawater allowed tentacles and cnidae to maintain stinging capacity and that, during attempts at removal, this allowed for additional stinging away from the initial contact site (Figure 3). Thus, TSBAA observations for seawater are divergent from the “inert” results found by TSAs, thus demonstrating that TSA results are not predictive of results in functional assays.

**Figure 4:**
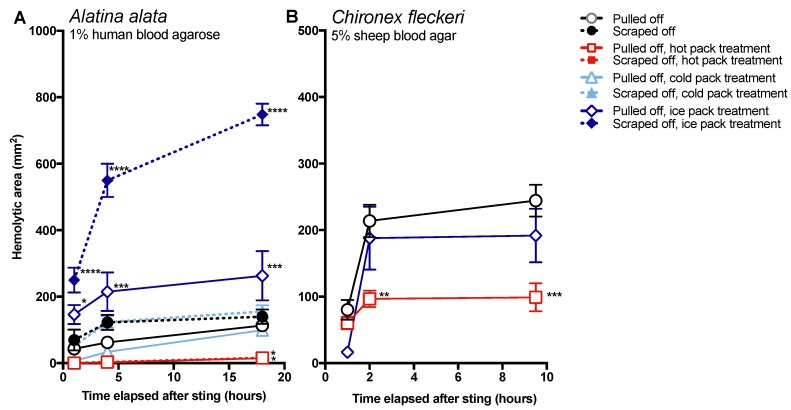
Size of hemolytic zone over time when tentacles were removed by scraping with a credit card (closed markers) or plucking with forceps (dashed lines) and treated after removal for 20 min for *A. alata* and 45 min for *C. fleckeri* with hot packs (red lines with squares), cold packs (light blue lines with triangles), ice packs (dark blue lines with diamonds), or ambient body temperature (black lines with circles). Significant differences between the treatments were found (two-way repeated measures ANOVA); significant post hoc Fisher’s LSD multiple comparisons to pulled off tentacles are denoted with asterisks: * *p* < 0.05, ** *p* < 0.01, *** *p* < 0.001, **** *p* < 0.0001. For *A. alata* (**A**), treatment course accounted for 70.67% of the variation (*p* < 0.0001), while time after sting accounted for 6.141% (*p* < 0.0001), the interaction between time and treatment for 10.76% (*p* < 0.0001), and individual variation for 9.257% (*p* < 0.0001). Scraping led to increases in hemolytic zone size when compared to pulling off the tentacle with tweezers, however, the most dramatic increases in lysis were caused by ice-pack treatment, which more than doubled the lytic zone size at 18 h, even in the absence of scraping. The use of hot packs significantly reduced the resultant size of the hemolytic zone after 18 h, regardless of whether tentacles were removed by scraping (*p* = 0.0167) or pulling (*p* = 0.016), while no significant differences were found between cold and room-temperature treatments of the same removal method. Interestingly, while the efficacy of heat was also seen with *C. fleckeri* (**B**, red line), the exacerbation with ice was not (**B**, blue line).

We also investigated the effect of temperature treatment on venom activity. Again, scraping had a significant deleterious impact in that larger areas of lysis were observed when scraping was followed by exposure to normal body temperature (37 °C) treatment (Figure 4, black dashed versus solid lines). However, nearly all of this functional metric of venom activity could be abolished if tentacle removal was immediately followed by 20 min of 45 °C hot pack application (Figure 4, red lines). In contrast to the potent inhibitory effect of hot pack application, cold packs (10 °C), often recommended for pain from cnidarian stings, were statistically indistinguishable from treatments of the same removal methods exposed to normal body temperatures (Figure 4, light blue lines compared to black lines), and therefore did not counteract the negative effects of scraping. Ice packs severely exacerbated *A. alata* stings, more than doubling the hemolytic area size after 18 h (Figure 4, dark blue lines). This was less evident in assays of *C. fleckeri* tentacles, which, due to the extreme bioactivity of the venom, led to almost complete lysis under the area of the tentacle even in the room temperature-incubated plates.

**Figure 5:**
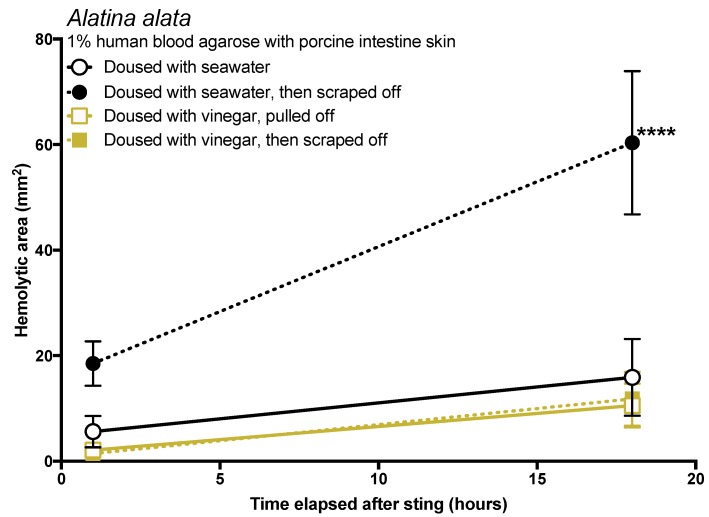
Size of venom-induced hemolytic zone over time when tentacles were doused with either vinegar (tan lines) or seawater (black lines) and removed by scraping with a credit card (closed markers, solid lines) or plucking with forceps (open markers, dashed lines). Significant differences between the treatments were found (two-way repeated measures ANOVA). Dousing solution accounted for 37.19% of the variation (*p* < 0.0001), while time after sting accounted for 15.43% (*p* = 0.001). The hemolytic zone size of seawater-doused, scraped tentacles was significantly greater than non-scraped seawater-doused tentacles or vinegar-doused tentacles (post hoc Fisher’s LSD, all comparisons *p* < 0.0001), regardless of removal strategy.

We further sought to determine whether vinegar dousing (without tentacle removal; seawater used as a solution control) has a significant impact on the amount of venom delivered if tentacles are then scraped off. Without vinegar dousing, scraping caused a massive increase in the functional metric of discharged venom, measured by an increased area of red blood cell lysis, of more than 3 times the size of the vinegar-rinsed samples. Increasingly significant differences between treatments were observed as the time course progressed (two-way repeated measures ANOVA, *p* < 0.0001; Figure 5). Vinegar-treated tentacles exhibited almost no blood agarose hemolysis (the metric of venom injection), and scraping did not lead to an increased hemolytic area if tentacles were first doused with vinegar for a period of 30 s.

### 2.3. Preliminary Investigation of Other Tentacle and Shed Cnidae Removal Aids

As shaving cream and baking soda are often recommended to aid in scraping, we also evaluated whether they inhibit or induce cnidae discharge in *A. alata* tentacles using the TSA. In addition, we examined dilutions of vinegar to determine if diluted vinegar is able to prevent cnidae discharge to the same extent as full-strength vinegar. Significant differences were found between the tested solutions (two-way ANOVA *p* < 0.0001). Concurrent with previous studies [16,17], 100% vinegar irreversibly inhibited cnidae discharge, even when pressure was applied to the tentacle (Table 1). However, as that vinegar was diluted with seawater, it lost its ability to prevent discharge. Shaving cream and baking soda slurries induced significant discharge and failed to prevent cnidae discharge from pressure.

## 3. Discussion

Effective and safe removal of adherent tentacles and the microscopic, intact, shed cnidae left on skin after tentacle contact are of paramount importance in the management of life-threatening cubozoan stings. In this study, scraping tentacles with a credit card was inefficient at removing cnidae, causing an increase in discharge while failing to remove many adherent cnidae (Figure 1). Scraping also caused increases in venom load, as evidenced by larger hemolytic zones in blood agarose (Figure 2, Figure 3, Figure 4 and Figure 5). These data suggest that tentacle removal by scraping has the potential of causing immense harm, and thus should no longer be recommended.

The data in this study indicated that tentacle removal by scraping can have a significant impact on sting severity. There are three possible ways that scraping could increase the amount of venom injected: (1) scraping could cause the tentacle to drag or roll, exposing a greater number of cnidae to the skin so they can sting; (2) scraping could apply pressure to tentacle cnidae and shed cnidae, inducing discharge; or (3) pressure from scraping could cause already-discharged cnidae to exude more venom, as has been speculated [41]. The first two were observed experimentally in this study but are difficult to discern in actual stings, as both would appear as an increase in discharged cnidae on the skin surface. In either case, we would expect that the application of a solution which inhibits cnidae discharge to prevent scraping-induced venom injection. In vitro studies in the past have strongly supported the use of vinegar (dousing the area for at least 30 s) prior to any removal attempts because it inhibits cnidae discharge [17,18], and case series have also supported its use [48]; our results concur with these findings, as vinegar not only prevented cnidae discharge in vitro (Table 1), dousing tentacles with it prior to removal significantly reduced the amount of venom delivered in our blood agarose model (Figure 5). Some have suggested that the addition of vinegar increases the venom exuded from already-discharged cnidae [49]. We found no evidence for this in our data. If the application of vinegar caused an increase in venom deposition from already-discharged cnidae, then we would have expected an increased zone of hemolysis after its application. Instead, the opposite occurred. Thus, we conclude that there is no evidence that vinegar application induces further venom release from discharged cnidae, and that vinegar should be considered a safe rinse solution for cubozoan stings. We also found that dilutions of vinegar progressively led to a reduction in the ability to prevent cnidae discharge (Table 1), thus vinegar should not be diluted prior to its use.

Most peer-reviewed and medical texts make it clear that scraping or tentacle removal should occur after inactivation of cnidae (specifically recommending vinegar in most cases) [20,21,22,23,24,25,26,27,28]; however, in the modern world of smartphones and social media, the general public does not turn to peer-reviewed journal articles or medical textbooks for information, especially in the heat of the moment during a painful event like a sting. Lay articles are less careful with their recommendations; some of the top Google search results for “What to do if stung by a jellyfish” do not specify proper inhibition of cnidae discharge prior to tentacle removal, including well-known and trusted sites. For example, WebMD says that after removing a victim from the water, you should “Wash the area with seawater to deactivate stinging cells. Or you can remove tentacles by scraping with a credit card or other plastic object” [29]. It should be noted that there is no evidence that seawater rinse can “deactivate stinging cells” and, to the contrary, this effort only serves to spread intact cnidae onto surrounding skin. Only after this advice is vinegar dousing mentioned. Similarly, the Mayo Clinic says to “remove stingers” by rinsing with seawater or “gently scraping off the stingers with the edge of an ID card or a credit card”, and then, after stinger removal, suggests vinegar rinsing [31]. Moreover, a visually appealing infographic [35] that was widely shared and has tens of thousands of views [36] depicts rinsing with seawater, scraping, and then applying vinegar. If a victim or someone helping them acted as these sites advise, they would likely worsen the sting (sees Figure 2, black solid versus dotted lines) and in the case of severe cubozoan envenomations, this unvalidated advice could actually cost lives. Some sources suggest applying shaving cream or baking soda to the adherent tentacle(s) and scraping with a credit card or razor blade if vinegar is unavailable or as an alternative to vinegar [37,38]. According to our results, the use of shaving cream and baking soda slurries should be considered suspect at best, as they do not inhibit cnidae discharge and cause undischarged cnidae to fire (Table 1).

Exactly where the suggestion to scrape away tentacles and/or cnidae came from is unclear. It is possible that it originated from co-opting the recommended first aid for removal of hymenopteran stingers [50], mistakenly assuming it would be helpful in removing cnidae capsules and tubules. In our study, scraping with a credit card with moderate force did not remove cnidae tubules already penetrating into our skin substitute, and did not even completely remove undischarged cnidae on the skin surface (Figure 1). Thus, we find it unlikely that scraping a sting site removes the penetrant foreign material. Or, scraping may have emerged from early tentacle removal recommendations, which consist of covering tentacles with dry, powdery substances, such as flour or even sand, to “coat” them before the they are scraped away with a knife or stick [51] or rubbed off with a towel or cloth [52]. In these early mentions, it was made clear that preventing further tentacle contact is paramount, as tentacle manipulations could be injurious and dangerously counterproductive; for example, Barnes [51] specifically states “ineffectual plucking and rubbing cause further discharge of nematocysts, greatly aggravating the injury” (p. 325) and Cleland and Southcott [52] similarly note that “clearly any action which will enhance the degree of contact of the tentacle with the skin is highly undesirable, as it may cause further discharge of nematocysts”, noting that dry sand may be better than wet because it “is more likely to fill the space between the tentacle and the skin, and when the tentacle is rolled in attempts to remove it, a better protective layer would exist between it and the skin” (p. 167). It is possible that there is a substance that could be applied similarly to these which would work to separate the tentacle from the skin or prevent tentacle rolling, thus making scraping less dangerous, but the data in this study indicate that shaving cream and baking soda are not ideal for this because of their induction of cnidae discharge and failure to inhibit pressure-induced discharge.

Tentacle removal methods outside of scraping also impacted sting severity. As compared with removal with tweezers, tentacle removal by rinsing with seawater significantly increased the hemolytic zone size (a functional metric of increased venom load) in both species tested, while rinsing with ethanol modestly increased venom load in *A. alata* (Figure 2). Increased hemolytic zone size after tentacle dousing with ethanol would be consistent with solution-based studies, which have shown that the application of alcohols increases cnidae discharge (e.g., [16,17,18]). However, the increase in hemolytic zone size after seawater rinse was unexpected, as seawater is generally considered a “safe” rinse solution, as it does not cause cnidae discharge in in vitro studies (e.g., [16,17,18]). Upon examination of the blood agarose slides, it was clear that seawater allowed tentacle pieces, shed cnidae, and intact still fully active cnidae to be moved away from the initial site of contact, thus creating an opportunity for stinging beyond the original tentacle skin contact zone (Figure 3). These results call into question the use of “innocuous” or “do no harm” rinse solutions in the removal of adherent tentacles and cnidae, as they may inadvertently permit additional stinging and thus lead to increases in venom delivered into the victim. This somewhat counterintuitive finding also underscores the concept that rinse solutions should be limited to solutions that actively inhibit cnidae discharge, rather than those that are deemed “inert”.

While removal methods produced similar results in both species tested (Figure 2), there were intriguing differences between the two, which may indicate species- or family-specific differences. *Chironex* tentacles were more adherent, and could not be removed from our tissue models with seawater or vinegar alone. Instead, tentacles had to be plucked with tweezers after attempted rinses. The only solution which led to effective detachment of *C. fleckeri* tentacles was Sting No More^®^ Spray, which contains urea, magnesium sulfate, and copper gluconate in addition to vinegar. We hypothesize that in addition to preventing further discharge due to the inclusion of vinegar, this weak acid with chaotropic urea in Sting No More^®^ Spray effectively dissolves glutinous substances released by cnidae or gland cells; further investigation of the mechanism by which Sting No More^®^ Spray releases adherent tentacles is warranted.

The data in this study indicate that post-removal care can greatly impact sting severity. There is much debate about whether heat is recommended in the treatment of cnidarian stings, although the preponderance of evidence supports its use [14]. Our findings strongly support the use of heat, as the application of hot packs was able to significantly reduce the activity of injected venom, to the point of completely counteracting the negative effects of scraping for *A. alata* (Figure 4). Heat application significantly reduced hemolytic area size for *C. fleckeri* stings, but not quite as effectively as for *A. alata* stings, which may indicate differential thermal tolerances of the venom toxins from the two species. Instead of alleviating the sting, the application of ice led to a dramatic increase in *A. alata* sting severity, more than doubling the size of the hemolytic zone at 18 h, even in the absence of scraping (Figure 5). Stings where tentacles were scraped off and then treated with ice for 20 min had hemolytic zones more than 6.5 times as large as stings where tentacles were pulled off and not treated with any temperature treatment. The application of cold packs (which only cooled to ~10 °C) did not have this effect, and were statistically indistinguishable from untreated stings receiving the same removal method. Ice pack treatment did not lead to statistically significant increases in the hemolytic zones of *C. fleckeri* stings; however, we would caution that the lack of increased hemolysis reflected a limitation of the sting model and conditions used. Because of the *Chironex* tentacle cnidae packing density and venom potency, near-complete lysis of the 5 mm deep blood agar under the tentacle was seen even at room temperature; therefore, it was not possible to discern increases in lysis. As a remote field site and the seasonal availability of the animals limited our work with *Chironex*, we were unable to conduct further experiments to more completely examine the effects of ice. To further examine distinctions between temperature effects, we plan to explore shorter sting durations and even thicker blood agar preparations. Future work on the effects of ice in chirodropid stings is essential, as there are reported deaths where ice was used [43]. Even if ice truly does not severely exacerbate all cubozoan stings, we would still caution that the use of ice is ineffective at best and disastrous at worst, and thus should be no longer considered a viable treatment option for cubozoan stings, while hot packs or hot water immersion should be universally applied in all cubozoan stings.

## 4. Conclusions

As the number of cnidae discharging in a cubozoan sting site represents a small fraction of the packed cnidae along the tentacle surface [16], first-aid steps taken immediately after a sting—particularly when tentacles and shed cnidae may be still adherent—can have dramatic effects on venom load and thus clinical outcomes.

For the cubozoans tested, we found that common recommendations—including rinsing the area with seawater, scraping away tentacles, and the application of ice packs—can severely exacerbate venom-induced hemolysis, a metric of sting sequelae. Our results suggest that these practices be immediately discontinued, and instead support rinsing with Sting No More^®^ Spray, vinegar, or, if no vinegar is available, that tentacles be removed by careful plucking. Tentacle removal is then best followed by the application of 45 °C heat or 45 °C hot water immersion for 45 min.

While our results provide robust support for this revised set of recommendations, further investigations are warranted. First, while it was clear that for both *A. alata* and *C. fleckeri*, ice packs were not helpful in reducing venom activity, experimental constraints limited our ability to conclusively determine whether ice packs could exacerbate *Chironex* stings. Thus, we caution against a benign interpretation of the *Chironex* results. However, if the lack of severe exacerbation by ice in *C. fleckeri* is validated by further study, then the divergence between the two species in their reaction to ice treatment could represent family-level differences in venom composition or tentacle behavior, or could reflect differences between species based on environmental factors (such as ambient seawater temperature). Second, comparisons should also be made to the other major cnidarian classes, the Schyphozoa and Hydrozoa. It is imperative to systematically compare removal methods and temperature effects to test for species-specific, as well as geographic-specific, differences to inform on management of jellyfish stings. Third, further work could also examine a greater diversity of recommended first-aid measures. It would be particularly interesting to examine whether traditional knowledge may provide potentially effective sting mitigation methods.

## 5. Materials and Methods

### 5.1. Chemicals

The chemicals used in all assays are as follows: freshwater (tap water), seawater (locally collected, 0.45 μm filtered), white vinegar (Bakers and Chefs CJ314, SAM’s West Inc., Bentonville, AR, USA), shaving cream (Gillette Regular Foam, Proctor and Gamble, Boston, MA, USA), ethanol (Pharmco-Aaper, Brookfield, CT, USA), Sting No More^®^ Spray (Alatalab Solutions LLC, Honoulu, HI, USA).

### 5.2. Animal Collections and Husbandry

Live *C. fleckeri* were collected by hand at Mapoon, Queensland, Australia (12°0′59.68″ S 141°54′15.83″ E) and kept in buckets with ample seawater until use in experiments (<2 days post-collection). Live *A. alata* were collected by hand from Waikiki Beach, Hawaii, USA (21°16′19.57″ N 157°49′25.04″ W) during their monthly spawning aggregations, and kept in buckets with ample seawater until use in experiments (<2 days post-collection).

### 5.3. Model Skin-Scraping Test

To replicate the circumstances of a natural sting, a sterile porcine intestine “skin” was placed on Saran Wrap (cling film) and wrapped around the arm of a volunteer. Skin preparation is described in detail in [16], but briefly: porcine intestines were cut into sheets and rinsed with 50 mM saline. Sheets were sterilized using 5% hydrogen peroxide, 10% ethanol solution in 50 mM saline for several hours. Sterile sheets were then rinsed with 110 mM saline, and sterile, warmed lanolin was worked into the intestine to create a water-resistant barrier. Five-centimeter lengths of freshly cut tentacles were applied to the skin and allowed to sting for 10 min. After the sting, tentacles were removed by either (a) pulling them off with tweezers or (b) scraping them off with a credit card using moderate pressure, as felt comfortable and natural to the volunteer. The skins were then examined using a dissecting microscope at 10× magnification (Olympus model SZX16, Olympus Corporation, Tokyo, Japan), and images were taken of three areas under the tentacle. Counts of discharged and undischarged cnidae were performed in ImageJ (National Institutes of Health, Bethesda, MD, USA).

### 5.4. Tentacle Solution Assay (TSA)

To test for the induction of discharge by shaving cream, freshly cut tentacles from live specimens (length ~1 cm; used <10 min after cutting) were placed on clean, dry microscope slides. The tentacle was then covered in the test solution (100 µL) or shaving cream (~2 cm^3^). After 1 min of incubation, the bulk of the test solution or cream was carefully removed and a cover slip was gently placed over the tentacle. To test whether solutions or shaving cream inhibited pressure-induced discharge, freshly cut tentacle pieces (length ~1 cm) were placed on clean, dry microscope slides and allowed to incubate for 1 min in the test solution/cream. A cover slip was added, and then the slide-tentacle-slip sandwich was pressed by hand for 30 s. All photos were taken after 10 min using an inverted microscope at 10× magnification (Olympus model CKX41SF, Olympus Corporation, Tokyo, Japan). Counts of discharged and undischarged cnidae were performed in ImageJ (National Institutes of Health, Bethesda, MD, USA).

### 5.5. Blood Agarose Assays

Functional venom delivery and activity was determined using blood agarose envenomation models [16]. Two variations of substrate were used: human blood agarose and sheep’s blood agar. For the human blood agarose, live human red blood cells (RBCs) were collected from normal donors (approved protocol CHS#12561, University of Hawaii Committee on Human Studies) and suspended in low melting point agarose to create an envenomation model in which venom activity is measured by hemolysis. The agarose was comprised of 1% RBC and 1.5% Nusieve GTG Agarose (Lonza, Rockland, ME, USA) in modified RPMI (“YRPMI”: 23.81 mM NaHCO_3_, Fisher; 102.67 mM NaCl, BDH; 5.37 mM KCl, Fisher; 0.41 mM MgSO_4_·7H_2_O, Fisher; 25 mM HEPES, Fisher; 6.67 mM NaH2PO_4_, Fisher; 0.42 mM Ca(NO_3_)_2_·4H_2_O, Fisher). For the sheep’s blood agar, standard 5 mm thick microbiology plates were used (Remel™ Contact Blood Agar Plates, TSA w/5% Sheep Blood, Fisher).

Blood agarose lengths (75 mm × 25 mm × 2 mm) or squares (25 mm × 25 mm × 2 mm) were placed on glass slides. Slides were placed in specialized holders whose height was equal to that of the agarose so that the surface of the agarose could be scraped without destroying its integrity. Agarose substrates were exposed to ~1.5 cm of freshly cut tentacle for 5 min (*A. alata*) or 2 min (*C. fleckeri*), after which the tentacle was either removed using tweezers (no scrape control), scraping with a credit card, or rinsed off using a test solution.

To evaluate the effects of scraping, two experimental setups were used; in both cases, treatments were assigned at random using a random number generator, and the conductor was blinded as to the treatment until after the application of tentacles. The first employed a sterile porcine intestine “skin” over the blood agar/agarose (“TSBAA” [16]) to allow for the application of test solutions. Using this set up, the comparison between scraping and rinsing was performed; tentacles were placed at one end of the substrate for the sting duration (see above), and then tilted to a 75-degree angle for tentacle removal. Tentacles were pulled off with tweezers, scraped off with a credit card, or rinsed using a test solution (seawater, tap water, vinegar, Sting No More^®^ Spray, or 95% ethanol). If the solution failed to remove the tentacle after 30 s of application, then the tentacle was pulled off manually; a total of 6 replicates were conducted for each removal method. To study the effects of dousing with vinegar, seawater or vinegar was applied to *A. alata* tentacles 2 min into the 5 min sting. Both scraping and the solution used were tested using a fully factorial approach (*n* = 6 for all).

To evaluate the efficacy of post-sting temperature applications on venom activity, a similar model without a skin (“TBAA” [16]) was used. One-time use cold packs (CVS Pharmacy Inc., Woonsocket, RI, USA), ice packs (cubed or crushed ice in Ziploc bags), reusable hot packs (45–50 °C at the surface, long lasting over 45 min; Emergency Zone LLC, Orem, UT, USA), or no treatment (left at ambient room temperature) were applied for 20 min to scraped and not-scraped agar/agarose using a fully factorial approach (*n* = 6 for all). For both experiments, controls without stinging activity were also run, and replicates were incubated at 37 °C overnight with images taken at 1, 4, and 18 h.

Images were recorded using a dissecting microscope (Olympus model SZX16, Olympus Corporation, Tokyo, Japan) or a DSLR camera (Nikon D800, Nikon Inc., Melville, NY, USA). The area of the zone of hemolysis was calculated using ImageJ. Briefly, scale was set using known object widths and subsections (15 mm × 5 mm for squares, 50 mm × 20 mm for lengths, 65 mm × 18 mm for plates) were taken for analysis to remove edge effects. Control slides and unstung areas were used to determine the color threshold for >80% hemolysis, and the total area of the hemolytic zone was taken directly from the “analyze particles” function. Hemolytic zone was evaluated as the area of the subsection exhibiting >80% hemolysis. Outliers were defined using the median absolute deviation (MAD) method detailed in [53], with the level of decision set conservatively at 3; any replicates which were outliers at all time points were removed. All ANOVA analyses and post hoc multiple comparisons were conducted in GraphPad Prism vers. 6.0 (GraphPad Software, Inc., La Jolla, CA, USA).

## Figures and Tables

**Table 1:** Induction or inhibition of cnidae discharge by shaving cream and vinegar. The solutions significantly differed in cnidae discharge (two-way ANOVA, *p* < 0.0001). Asterisks indicate significant difference from seawater in the same column according to post hoc Fisher LSD tests; crosses indicate solution of vinegar difference from 100% vinegar within the same column. Shaving cream and both baking soda slurries significantly increased discharge. Vinegar solutions with 25%–100% vinegar prevented pressure-induced cnidae discharge, however, the more diluted the vinegar, the less protective it was against this discharge.

Solution	Solution-Only Cnidae Discharge (%)	Pressure-Induced Cnidae Discharge (%)
Seawater	01.80 ± 1.80	47.20 ± 4.70
Vinegar	00.73 ± 0.32	05.29 ± 2.46 *
75% Vinegar in Seawater	01.36 ± 0.25	11.44 ± 1.22 *
50% Vinegar in Seawater	00.00 ± 0.00	16.11 ± 3.92 *^,†^
25% Vinegar in Seawater	00.44 ± 0.26	29.02 ± 1.29 *^,†^
10% Vinegar in Seawater	00.56 ± 0.32	47.96 ± 1.92 ^†^
Shaving Cream	17.36 ± 5.00 *	36.42 ± 5.60
Baking Soda (1:1 with Seawater)	50.38 ± 3.70 *	53.88 ± 2.31
Baking Soda (3:1 with Seawater)	38.46 ± 6.36 *	48.58 ± 0.42

## Abbreviations

The following abbreviations are used in this manuscript:

TSBAA	Tentacle Skin Blood Agarose Assay
TBAA	Tentacle Blood Agarose Assay
TSA	Tentacle Solution Assay
Fisher’s LSD	Fisher’s Least Significant Difference
